# Serum Interleukin-6 and Interleukin-10 levels in people living with HIV on short-term and long-term antiretroviral therapy: a comparative cross-sectional study with HIV-negative controls

**DOI:** 10.1186/s12879-026-13859-6

**Published:** 2026-06-27

**Authors:** Adekunle Ayomide Adegoke, Chizaram Onyeaghala, Abel Oluwaseun Olojede, Olufemi G. Omitola, Chijioke A. Nwauche

**Affiliations:** 1 Department of Haematology, Blood Transfusion and Immunology, Faculty of Basic Clinical Sciences, University of Port Harcourt, Port Harcourt, Rivers State Nigeria; 2 Department of Oral Pathology and Oral Biology, Faculty of Dentistry, University of Port Harcourt, Rivers State Port Harcourt, Nigeria; 3 Department of Internal Medicine, University of Port Harcourt Teaching Hospital, Rivers State Port Harcourt, Nigeria

**Keywords:** Interleukin-6, Interleukin-10, Cytokine profile, People living with HIV, Antiretroviral therapy, Nigeria

## Abstract

**Background:**

Antiretroviral therapy (ART) suppresses HIV replication and partially restores immune function, but immune recovery is often incomplete. Interleukin-6 (IL-6) and Interleukin-10 (IL-10) reflect opposing sides of the immune response, pro-inflammatory and regulatory, respectively. They may provide insight into the degree of residual immune dysregulation in people living with HIV (PLHIV) on treatment. This study aims to determine serum IL-6 and IL-10 levels in PLHIV on ART compared to HIV-negative controls.

**Methods:**

A comparative cross-sectional study was conducted at the HIV Care and Treatment Clinic of the University of Port Harcourt Teaching Hospital (UPTH), Nigeria. Eighty-one participants were enrolled: 63 were living with HIV (27 on short-term ART [less than six months], 36 on long-term ART [two years or more]), and 18 HIV-negative controls. Serum IL-6 and IL-10 were quantified by enzyme-linked immunosorbent assay (ELISA). Group differences were assessed using one-way analysis of variance (ANOVA), Tukey HSD post hoc tests, and independent samples t-tests. The influence of age and sex on cytokine levels was examined by analysis of covariance (ANCOVA).

**Results:**

IL-6 was 7.35 ± 0.17 pg/ml in the short-term ART group, 6.38 ± 0.12 pg/ml in the long-term ART group, and 5.65 ± 0.15 pg/ml in controls. All three pairwise comparisons were statistically significant (*p* < 0.05). IL-10 was 6.42 ± 0.15 pg/ml, 6.21 ± 0.11 pg/ml, and 5.81 ± 0.22 pg/ml across the same groups respectively. Only the comparison between the short-term ART group and controls was significant for IL-10. Age and sex had no statistically significant effect on either cytokine.

**Conclusion:**

IL-6 declines with longer ART but remains elevated above control levels even after years of treatment, indicating persistent residual inflammation. Interleukin 10 is elevated in treated individuals compared with controls but does not track ART duration. These findings support the value of cytokine profiling as a supplementary tool for monitoring immune status in ART-treated PLHIV in a Nigerian clinical setting.

**Supplementary Information:**

The online version contains supplementary material available at https://doi.org/10.1186/s12879-026-13859-6.

## Introduction

Human immunodeficiency virus (HIV) infection remains a leading global public health challenge, with sub-Saharan Africa bearing the greatest burden [1]. Antiretroviral Therapy (ART) has dramatically improved survival by suppressing viral replication, increasing CD4 + T-cell counts, and reducing the incidence of acquired immunodeficiency syndrome-defining illnesses [2]. However, full immune recovery does not occur uniformly. A significant proportion of ART-treated individuals continue to show residual inflammation, immune dysregulation, and elevated risk of non-AIDS conditions including cardiovascular disease, metabolic disorders, and certain cancers [3].

Conventional markers of ART response, namely CD4 + T-cell count and plasma viral load, do not capture the full complexity of immune activation. Clinicians in resource-limited settings such as Nigeria often lack tools to identify patients who maintain residual immune activation despite apparent virologic control [4]. This creates an important monitoring gap that cytokine-based markers may help address.

Interleukin 6 (IL-6) and Interleukin 10 (IL-10) represent the two opposing arms of the cytokine response most relevant to HIV-associated immune dysregulation. Interleukin 6 is a pro-inflammatory cytokine produced by monocytes, macrophages, and T cells in response to viral stimulation and immune activation. It promotes acute-phase responses, drives T-cell differentiation, and has been linked to non-AIDS comorbidities and excess mortality in people living with HIV (PLHIV) in longitudinal cohort studies [5, 6]. Specifically, higher IL-6 levels have been independently associated with non-AIDS-related death, cardiovascular disease, and non-AIDS-defining malignancies in ART-treated adults in other population predominantly in North American and European settings [7, 8]. By contrast, IL-10 is an anti-inflammatory cytokine that acts as a brake on immune activation and limits immune-mediated tissue damage. Its sustained elevation in HIV infection has been associated with impaired antiviral immunity and viral persistence [9]. Together, these two cytokines reflect the balance between immune activation and regulation, making them biologically meaningful and clinically informative candidates for supplementary immune monitoring in PLHIV.

Most studies examining these cytokines in HIV have been conducted in high-income North American or European settings [10, 11], where genetic backgrounds and treatment contexts differ substantially from those in high-burden sub-Saharan Africa, particularly West Africa. De Medeiros et al. and Obeagu et al. have directly compared cytokine levels across short-term and long-term ART with HIV-negative controls in Nigerian populations [12, 13]. Short-term ART is defined as less than six months of treatment, a period of active immune reconstitution and heightened inflammatory activity. Long-term ART is defined as two years or more of treatment, a stabilised treatment phase in which cytokine patterns are expected to plateau [14, 15]. These thresholds are consistent with established immunological milestones described in the HIV literature.

This study aims to measure serum IL-6 and IL-10 levels in PLHIV receiving short-term and long-term ART, compared with HIV-negative controls. The findings are intended to provide locally generated immunological data to support HIV monitoring and clinical decision-making in Nigeria.

## Materials and methods

### Study design and setting

This was a comparative cross-sectional study conducted at the HIV Care and Treatment Clinic of UPTH, Rivers State, Nigeria. A cross-sectional design was adopted because the study objective required simultaneous measurement of cytokine levels and clinical variables across defined participant groups at a single point in time, without follow-up or longitudinal tracking. The clinic operates one of the largest HIV treatment programmes in South-South Nigeria. 

### Study population, recruitment and duration 

Between October 6 and November 8, 2025, a total of 81 participants were enrolled using a combination of simple random, consecutive, and purposive sampling techniques, and data were collected with a pretested, structured, interviewer-administered questionnaire (Supplementary File 1). Participants were allocated into three groups in a 3:4:2 ratio: Group A (short-term ART, *n* = 27), Group B (long-term ART, *n* = 36), and Group C (HIV-negative controls, *n* = 18). The 3:4:2 ratio was adopted to ensure adequate representation of the long-term ART group, who formed the primary focus for evaluating sustained immune modulation and who were more readily available within the clinic population during the recruitment period. This allocation maintained meaningful representation across all three groups for comparative analysis.

For Group B, two participants were selected by simple random sampling on each clinic day over five weeks, Mondays through Thursdays, when the clinic operated, to minimise recruitment bias. Group A consecutively recruited all eligible short-term ART patients presenting during the five-week recruitment period. Group C was recruited by purposive sampling from UPTH staff and voluntary blood donors. These individuals were selected because they were readily accessible, routinely screened for HIV as part of occupational health and blood donor screening protocols, and were considered apparently healthy adults without known chronic or inflammatory conditions. Human immunodeficiency virus-negative status was confirmed by standard screening prior to enrolment.

The questionnaire captured demographic data, baseline clinical characteristics (including ART regimen), and laboratory information. In particular, ART regimen data for participants living with HIV were classified into two pharmacological classes. INSTI-based regimens comprised TDF/3TC/DTG and ABC/3TC/DTG, and PI-based regimens comprised TDF/3TC/ATV/r, TDF/3TC/DRV/r, and ABC/3TC/ATV/r. 

### Eligibility criteria

Inclusion criteria for Group And B were: a confirmed HIV diagnosis, current ART use at the UPTH HIV clinic, ART duration of less than six months (Group A) or two years or more (Group B), and age 18 years or older. For Group C, inclusion required confirmed HIV-negative status and age 18 years or older.

Exclusion criteria for all groups included: age below 18 years; active opportunistic infections at enrolment, such as tuberculosis, cryptococcal meningitis, or Pneumocystis pneumonia; active co-infections, including hepatitis B or C; pregnancy; use of immunomodulatory therapy; active autoimmune conditions; and history of major surgical procedures within the past six months: and comorbidities, as they can alter inflammatory or immune parameters. 

### Sample size calculation

Sample size was calculated using the Kish (1965) formula for prevalence studies: *n* = (Z^2^ x P x (1-P)) / d^2^, where Z = 1.96 (95% confidence level)[16], *P* = 0.039 (HIV prevalence in Rivers State, Nigeria [17]), and d = 0.05 (margin of error). This yielded a minimum sample of 58 participants. An 8% attrition allowance was added to account for potential unusable samples, giving 63 HIV-positive participants. Eighteen HIV-negative controls were added at a 7:2 ratio, giving a total of 81 participants allocated in a 3:4:2 ratio across the three groups. 

### Operational definition of terms

Short-term ART: This refers to people living with HIV who have been on antiretroviral therapy for fewer than six months [14].

Long-term ART: This refers to people living with HIV who have been on antiretroviral therapy for more than six months [15].

Viral suppression is defined as a plasma HIV RNA level below 1,000 copies/ml, consistent with WHO-defined treatment success criteria [18]. 

Undetectable viral load refers to a plasma HIV RNA level below 50 copies/ml, indicating full virological suppression below the lower limit of quantification of standard laboratory assays [19].

Low-level viremia is defined as a detectable plasma HIV-1 RNA viral load of 50 copies/ml or higher but below 1,000 copies/ml. 

Virally non-suppressed refers to a plasma HIV-1 RNA viral load of 1,000 copies/ml or higher, indicating failure of ART to achieve adequate virological control [18]. 

An integrase strand transfer inhibitor-based regimen is an antiretroviral therapy regimen that uses an integrase strand transfer inhibitor as the anchor drug. 

A protease inhibitor-based regimen is an antiretroviral therapy regimen that uses a protease inhibitor as the anchor drug. 

### Sample collection and cytokine analysis

Fresh venous blood samples were collected on the day of recruitment from all participants living with HIV for cytokine analysis and concurrent viral load measurements. The immunology scientist performed cytokine analysis, while the UPTH laboratory measured viral load as part of routine clinical care.

Five millilitres of venous blood were collected from each participant using plain vacutainer tubes. Samples were allowed to clot at room temperature for 30 min then centrifuged at 3,000 rpm for 10 min using an Eppendorf 5804R centrifuge. Serum was stored at -20°C in a Haier laboratory freezer in UPTH haematology department until analysis.

Serum IL-6 and IL-10 concentrations were quantified using commercial enzyme-liked immunosorbent assay (ELISA) kits from Eldere (Shanghai Ideal Medical Technology Co., Ltd.) under the supervision of a qualified medical laboratory scientist. Interleukin 6 standards were prepared at concentrations of 0, 3, 6, 12, 24, and 48 pg/ml. Interleukin 10 standards ranged from 0 to 40 pg/ml. All samples were run in duplicate. Optical density was measured at 450 nm using a BioTek ELx808 microplate reader within 15 min of stopping the reaction.

Viral load was measured concurrently by the UPTH laboratory as part of routine clinical care. Viral load did not apply to group C/control because they are HIV-negative.

### Statistical analysis

Data were analysed using IBM SPSS Statistics (version 20). Descriptive statistics were reported as mean ± standard error of the mean (SEM). Group differences in cytokine levels were assessed using one-way ANOVA with Tukey HSD post hoc tests for pairwise comparisons. A direct comparison of cytokine levels between the two INSTI- and PI-based ART groups was performed using an independent-samples t-test. ANCOVA was used to assess the influence of age and sex as covariates. One-way ANOVA was also used to compare cytokine levels across viral load classification categories.

Statistical significance was set at *p* < 0.05. Participants with reported values of less than 30 copies/ml or less than 20 copies/ml were recoded as 15 copies/ml and 10 copies/ml, respectively, for analytical purposes [19]. 

## Results

### Sociodemographic and baseline characteristics

Table 1 summarises the sociodemographic and baseline clinical characteristics of all three groups. Group A consisted of 40.7% males and 59.3% females, with ages ranging from 20 to 60 years and a mean of 35.00 ± 11.40 years. Group B contained 33.3% males and 66.7% females, with ages from 30 to 69 years and a mean of 48.08 ± 9.73 years. Group C had equal sex distribution (50% each) and a mean age of 34.06 ± 12.10 years. Mean ART duration was 4.85 ± 0.23 months in Group A and 91.42 ± 9.06 months in Group B. Mean viral load was 114.10 ± 51.20 copies/ml in Group A and 40.29 ± 24.12 copies/ml in Group B. All 27 participants in Group A were on INSTI-based regimens. In Group B, 28 participants (77.8%) were on INSTI-based regimens and eight (22.2%) were on PI-based regimens.

**Table 1:** Sociodemographic and baseline characteristics of study participants

Characteristic	Group A: Short-term ART (*n* = 27)	Group B: Long-term ART (*n* = 36)	Group C: Controls (*n* = 18)
Male, n (%)	11 (40.7%)	12 (33.3%)	9 (50.0%)
Female, n (%)	16 (59.3%)	24 (66.7%)	9 (50.0%)
Age range (years)	20 to 60	30 to 69	18 to 60
Mean age ± SD (years)	34.63 ± 11.10	49.33 ± 10.41	35.72 ± 12.23
Mean ART duration ± SEM (months)	4.85 ± 0.23	91.42 ± 9.06	N/A
Mean viral load ± SEM (copies/ml)	113.17 ± 51.27	39.46 ± 24.15	N/A
IL-6 (pg/ml, mean ± SEM)	7.35 ± 0.17	6.38 ± 0.12	5.65 ± 0.15
IL-10 (pg/ml, mean ± SEM)	6.42 ± 0.15	6.21 ± 0.11	5.81± 0.23

*SD = standard deviation; SEM = standard error of mean; N/A = not applicable*

### ANOVA and post hoc analysis for IL-6

One-way ANOVA showed a statistically significant difference in IL-6 levels across the three participant groups (F(2,78) = 28.480, *p* < 0.001). Table 2 presents the full ANOVA and post hoc results for IL-6. Tukey HSD post hoc analysis confirmed significant differences between all pairwise combinations. IL-6 was significantly higher in Group A than in Group B (mean difference = 0.97, *p* < 0.001), significantly higher in Group A than in Group C (mean difference = 1.70, *p* < 0.001), and significantly higher in Group B than in Group C (mean difference = 0.73, *p* = 0.004). The stepwise decline from short-term ART to long-term ART to controls confirms that IL-6 tracks ART duration and HIV status.

**Table 2:** T-test, ANOVA, and post hoc comparisons for IL-6 across study groups

Analysis	Comparison	Test statistic	*p*-value	Significant
Independent samples t-test	Short-term vs. long-term ART	Mean diff = 0.97 ± 0.20	< 0.001	Yes
One-way ANOVA	Between groups	F(2,78) = 28.480	< 0.001	Yes
Post hoc Tukey HSD	Short-term vs. long-term ART	Diff = 0.97	< 0.001	Yes
Post hoc Tukey HSD	Short-term ART vs. controls	Diff = 1.70	< 0.001	Yes
Post hoc Tukey HSD	Long-term ART vs. controls	Diff = 0.73	0.004	Yes

Significant = *p* < 0.05

### ANOVA and post hoc analysis for IL-10

IL-10 also showed a statistically significant difference across groups by one-way ANOVA (F(2,78) = 3.508, *p* = 0.035). However, Tukey HSD post hoc analysis revealed a more selective pattern. A significant difference was found only between Group A and Group C (mean difference = 0.61, *p* = 0.027). The comparison between Group A and Group B (mean difference = 0.20, *p* = 0.540) and between Group B and Group C (mean difference = 0.40, *p* = 0.162) were not statistically significant. Table 3 presents the full IL-10 results. The independent samples t-test also showed no significant difference in IL-10 between Groups A and B (mean difference = 0.20, *p* = 0.258).

**Table 3:** T-test, ANOVA, and post hoc comparisons for IL-10 across study groups

Analysis	Comparison	Test statistic	*p*-value	Significant
Independent samples t-test	Short-term vs. long-term ART	Mean diff = 0.20 ± 0.18	0.258	No
One-way ANOVA	Between groups	F(2,78) = 3.508	0.035	Yes
Post hoc Tukey HSD	Short-term vs. long-term ART	Diff = 0.20	0.540	No
Post hoc Tukey HSD	Short-term ART vs. controls	Diff = 0.61	0.027	Yes
Post hoc Tukey HSD	Long-term ART vs. controls	Diff = 0.40	0.162	No

Significant = *p* < 0.05

### Influence of age and sex

ANCOVA results are presented in Tables 4 and 5. For IL-6, neither age (F = 1.055, *p* = 0.308) nor sex (F = 1.151, *p* = 0.287) had a statistically significant independent effect. Interaction terms were also non-significant. Group assignment remained the only significant predictor (F = 30.500, *p* < 0.001 for the sex model; F = 23.516, *p* < 0.001 for the age model). For IL-10, age (F = 0.048, *p* = 0.828), sex (F = 0.001, *p* = 0.975), and all interaction terms were non-significant. Group was significant only in the sex model (F = 3.448, *p* = 0.037). These findings confirm that cytokine variation in this study was driven by HIV status and ART duration, not by demographic factors.

**Table 4:** ANCOVA results for the influence of age and sex on IL-6

Analysis component	Factor	F statistic	*p*-value	Significant
Effect of sex	Sex	F = 1.151	0.287	No
Effect of sex	Group	F = 30.500	< 0.001	Yes
Effect of sex	Group x sex	F = 1.493	0.231	No
Effect of age	Age	F = 1.055	0.308	No
Effect of age	Group	F = 23.516	< 0.001	Yes
Effect of age	Group x age	F = 0.243	0.785	No

*Significant = p < 0.05. Interaction terms reflect combined factor effects*

**Table 5:** ANCOVA results for the influence of age and sex on IL-10

Analysis component	Factor	F statistic	*p*-value	Significant
Effect of age	Age	F = 0.048	0.828	No
Effect of age	Group	F = 2.039	0.137	No
Effect of age	Group x age	F = 0.285	0.753	No
Effect of sex	Sex	F = 0.001	0.975	No
Effect of sex	Group	F = 3.448	0.037	Yes
Effect of sex	Group x sex	F = 0.715	0.493	No

*Significant = p < 0.05*

### Effect of ART regimen class on cytokine levels

As shown in Table 6, Among the 63 participants living with HIV, 55 (87.3%) were on INSTI-based regimens (TDF/3TC/DTG, *n* = 45, 71.4%; ABC/3TC/DTG, *n* = 10, 15.9%) and eight (12.7%) were on PI-based regimens (TDF/3TC/ATV/r, *n* = 5, 7.9%; TDF/3TC/DRV/r, *n* = 2, 3.2%; ABC/3TC/ATV/r, *n* = 1, 1.6%). No participant was on a nevirapine-based regimen. Mean IL-6 levels were 6.880 ± 0.126 pg/ml in the INSTI-based group and 6.209 ± 0.198 pg/ml in the PI-based group. Mean IL-10 levels were 6.280 ± 0.095 pg/ml in the INSTI-based group and 6.448 ± 0.249 pg/ml in the PI-based group. Independent samples t-tests showed no statistically significant differences in IL-6 levels (t = 1.971, *p* = 0.053) or IL-10 levels (t = -0.629, *p* = 0.532) between the two regimen classes. These findings indicate that the ART regimen class did not significantly influence IL-6 or IL-10 levels in this study.

**Table 6:** Distribution of antiretroviral therapy regimens and their percentages among participants on short-term and long-term ART

ART regimen class, *n* (%)	Group A: Short-term ART (*n* = 27)	Group B: Long-term ART (*n* = 36)
INSTI-based	27 (100.0%)	28 (77.8%)
PI-based	0 (0.0%)	8 (22.2%)

INSTI = integrase strand transfer inhibitor; PI = protease inhibitor

### Cytokine levels by viral load classification

Among the 63 HIV-positive participants with available viral load data, 55 (87.3%) had undetectable viral loads (less than 50 copies/ml), seven (11.1%) had low-level viremia (50 to 999 copies/ml), and one (1.6%) was virally non-suppressed (1,000 copies/ml or higher). Table 7 presents the mean IL-6 and IL-10 values by virological category.

**Table 7:** Viral load classification distribution and corresponding cytokine levels among participants living with HIV

Viral load category	Group A: Short-term ART (*n* = 27) *n* (%)	Group B: Long-term ART (*n* = 36) *n* (%)	Total (*n* = 63)	VL range (copies/ml)	IL-6 mean ± SEM (pg/ml)	IL-10 mean ± SEM (pg/ml)
Undetectable (<50)	20 (74.1%)	35 (97.2%)	55	10 to 43	6.708 ± 0.123	6.305 ± 0.099
Low-level viremia (50–999)	6 (22.2%)	1 (2.8%)	7	52 to 884	7.313 ± 0.311	6.193 ± 0.147
Non-suppressed (≥1,000)	1 (3.7%)	0 (0.0%)	1	1,246	7.968	6.869

SEM = standard error of mean

Single participant in the non-suppressed category; SEM not calculable.Viral load categories: undetectable, <50 copies/ml; low-level viremia, 50–999 copies/ml; non-suppressed, ≥1,000 copies/ml

## Discussion

This study found that serum IL-6 levels decline with longer ART but remain elevated above control levels even after years of treatment, indicating persistent residual inflammation despite viral suppression. Serum IL-10 levels are higher in treated individuals than in controls but do not correlate with ART duration. These findings support the value of cytokine profiling as a supplementary tool for monitoring immune status among ART-treated PLHIV in clinical settings.The study found a stepwise decline in serum IL-6 levels from short-term ART (7.35 ± 0.17 pg/ml) to long-term ART (6.38 ± 0.12 pg/ml) to HIV-negative controls (5.65 ± 0.15 pg/ml), with all pairwise comparisons reaching statistical significance. This pattern is consistent with the well-established progressive anti-inflammatory effect of sustained viral suppression [14, 15]. In the early months of ART, viral replication remains suppressed, and the immune system continues to respond to residual antigenic stimulation, microbial translocation resulting from gut mucosal damage sustained during untreated infection, and persistent lymphocyte activation that persists after initial immune reconstitution begins [18, 20]. Interleukin-6 production by monocytes and macrophages in response to these stimuli may account for the elevated levels observed in the short-term ART group. These findings align with those of Bordoni et al., who documented heightened cytokine activity during the early months of ART, and with Bastard et al., who characterized the inflammatory profile of ART-treated individuals across treatment stages [14, 15]. 

 The persistent elevation of IL-6 levels in the long-term ART group relative to HIV-negative controls is an important observation. Even after a mean treatment duration of 91.42 months, IL-6 levels remained significantly higher than those in apparently healthy HIV-negative individuals. This finding aligns with reports from high-income settings in the United States and Europe, as well as from Ethiopia in sub-Saharan Africa, showing that ART substantially reduces but does not fully normalise IL-6 levels in treated PLHIV [11, 21, 22]. The biological mechanisms most likely to account for this residual immune elevation include continued low-level activity of the latent HIV reservoir, persistent gut mucosal dysfunction with associated microbial translocation, and lasting reprogramming of monocyte and macrophage inflammatory responses [23, 24]. Latently infected resting CD4+ T cells and macrophages harbouring integrated proviral DNA undergo stochastic reactivation, producing low-level viral transcripts that stimulate innate immune sensors and maintain IL-6 secretion independently of plasma viral load [24, 25]. Eisele and Siliciano et al. demonstrated that the size of the latent reservoir correlates with residual immune activation markers in virally suppressed individuals [24]. Subsequent studies have provided supporting evidence for a reservoir-immune activation axis that persists across multiple years of effective ART [24, 26, 27]. The gut mucosal barrier does not fully recover under ART, and persistent impairment of tight junction integrity in the colonic epithelium has been documented in virally suppressed individuals, thereby sustaining the translocation of microbial products that activate monocyte-derived IL-6 production through Toll-like receptor signalling pathways [18, 28–31].

 The IL-6 profile observed in this study is biologically coherent and has been documented in virally suppressed populations in both high-income and low-income settings [11, 14, 22]. Confirming this pattern in a Nigerian population attending a tertiary HIV care clinic adds to the growing body of evidence that the immunological trajectory of ART-treated PLHIV may be broadly similar across geographic settings. The persistent IL-6 elevation observed in this study may reflect an inflammatory profile that, in previous longitudinal studies predominantly conducted in North American and European populations, has been associated with a range of long-term immune consequences among ART-treated PLHIV, including accelerated immunosenescence and chronic immune dysregulation [32–35]. Because study participants were screened to exclude active comorbidities and acute inflammatory conditions at recruitment, the elevated IL-6 observed here likely reflects intrinsic HIV-associated and ART-related immune activation rather than confounding by intercurrent illness, strengthening the interpretive value of the cytokine measurements.

Available literature on IL-6 levels in HIV consistently identifies persistently elevated IL-6 among virally suppressed individuals as an inflammatory marker associated with future risk of non-AIDS-related outcomes [7, 8, 36]. However, this study did not directly assess clinical outcomes, morbidity, or mortality. The observed cytokine elevations therefore reflect an inflammatory state that may have clinical significance over the longer term, consistent with mechanisms described in the literature, rather than evidence of current disease burden in this cohort. In a Nigerian clinical context, where access to advanced immunological monitoring is limited, the identification of persistently elevated IL-6 in apparently well-controlled ART-treated individuals may prompt clinicians to consider broader long-term clinical surveillance [4, 6]. 

The viral load classification analysis showed a trend toward higher IL-6 levels with increasing viral replication, from a mean of 6.708 ± 0.123 pg/ml in participants with undetectable viral loads to 7.313 ± 0.311 pg/ml in the low-level viremia group and 7.968 pg/ml in the single non-suppressed participant. Although ANOVA across these categories did not reach statistical significance (F(2,60) = 2.252, *p* = 0.114), likely due to the marked imbalance in group sizes, the stepwise pattern of concurrent elevations in IL-6 and viral load is biologically plausible. Even at an undetectable viral load, the mean IL-6 level of 6.708 pg/ml remained above the control mean of 5.650 pg/ml, consistent with reservoir-driven immune stimulation that persists independently of plasma viraemia [24, 26]. Participants with low-level viremia had the next highest mean IL-6 levels, which may reflect residual viral replication in pharmacological sanctuary sites such as lymph nodes and the central nervous system, where drug penetration may be reduced or suboptimal [37]. Low-level viremia has been associated with a higher risk of virological failure in other populations [37], and the trend toward higher IL-6 in this group may reflect the same residual viral activity that drives both immune stimulation and incomplete virological suppression. 

 Interleukin-10 showed a distinct, more restricted pattern. The only statistically significant pairwise difference was between the short-term ART group and controls. In contrast, comparisons between the two ART groups and between the long-term ART group and controls were not significant. This pattern indicates that IL-10 rises early in the setting of HIV infection and initial treatment, then reaches a plateau that is maintained regardless of ART duration. The biological explanation centres on IL-10's role as a broadly reactive anti-inflammatory cytokine induced through STAT3-dependent pathways in response to inflammatory stimulation [38]. IL-10 production is not tightly coupled to viral load, as some pro-inflammatory cytokines are; rather, it responds to ongoing immune activation from any source and remains elevated as long as inflammatory signals persist [38, 39]. Brockman et al. reported that IL-10 is upregulated in multiple immune cell types during HIV viremia and that its elevation partially reverses with successful ART but does not fully normalise [39]. The co-elevation of IL-10 alongside IL-6 across ART groups may reflect the biological coupling of pro-inflammatory and counter-regulatory responses. As IL-6-driven inflammation persists from reservoir activity and microbial translocation, IL-10 is sustained as a homeostatic response to limit immune-mediated tissue injury [38, 40]. An IL-10-producing CD8+ T-cell subset has also been described in chronic HIV infection that may contribute to this regulatory tone as an adaptive response to monocyte activation during sustained antigenic stimulation [40, 41].

 The absence of significant ANCOVA effects for age and sex on either cytokine confirms that the observed group differences are attributable to HIV infection status and ART duration rather than demographic variation. Although age-related increases in baseline IL-6 levels have been documented in healthy adult populations, sometimes termed inflammaging [33, 42, 43], the immunological impact of HIV infection and ART appears to override this demographic signal in this study [44]. Similarly, sex-based differences in cytokine responses have been reported in conditions including sepsis, cardiovascular inflammatory states, and experimental endotoxin challenge models [45, 46]. Still, these differences did not emerge in this study. This may reflect the dominant and relatively uniform effect of chronic HIV-associated immune activation across both sexes, which subsumes subtler hormonal influences on cytokine production [45, 46].

The ART regimen analysis also showed no statistically significant differences in IL-6 (t = 1.971, *p* = 0.053) or IL-10 levels (t = -0.629, *p* = 0.532) between INSTI- and PI-based regimens. The IL-6 level (*p* = 0.053) approached borderline significance, indicating a trend that a more powered study could resolve. PI-based regimens have been associated with higher systemic inflammatory markers, including interferon-alpha and C-reactive protein, compared with INSTI-based regimens, particularly in pregnant and early-treatment populations [47, 48]. Serrano-Villar and colleagues modelled the potential long-term immunological consequences of regimen-related IL-6 differences, suggesting that even modest differences in IL-6 levels between regimens may have meaningful implications for cumulative non-AIDS event risk over years of treatment [47]. In the present study, the predominance of INSTI-based regimens (TDF/3TC/DTG), reflecting contemporary Nigerian treatment guidelines favouring dolutegravir as first-line therapy [19, 49], and the small PI-based subgroup (*n* = 8) limit the statistical power to draw definitive conclusions. Similarly, Quiros-Roldan and colleagues reported no significant differences in cytokines between INSTI- and PI-based regimens in virally suppressed individuals [48]. Larger studies with more balanced representation of ART regimens are needed to better characterize this relationship in African populations.

This study has several limitations. First, the cross-sectional design captures cytokine levels at a single time point and therefore does not allow causal inference about the temporal dynamics of IL-6 and IL-10 changes across ART stages. Second, the single-centre location, coupled with nonprobability sampling during enrolment, may limit generalizability to other Nigerian or African settings. Third, ART regimen and treatment duration history relied on participants’ recall, which may have introduced recall or social desirability bias. Fourth, CD4 count data were not obtained from participants living with HIV, limiting the ability to correlate cytokine findings with an established marker of immune reconstitution. Additionally, clinical parameters of systemic inflammation, such as CRP, ESR, and ferritin, were not measured, preventing cross-validation of cytokine findings against complementary inflammatory markers. Fifth, the PI-based regimen subgroup was small, limiting the statistical power to detect modest differences in regimen-associated cytokines. Finally, the viral load classification analysis was substantially limited by group size imbalance, with 55 participants in the undetectable category compared with seven in low-level viremia and one non-suppressed participant. 

## Conclusion

This study demonstrated that serum IL-6 levels decline progressively from short-term to long-term ART in PLHIV but remain significantly elevated above levels observed in HIV-negative controls even after years of sustained treatment. Serum IL-10 levels were higher in treated individuals than in controls but did not correlate with ART duration. Declining IL-6 and elevated IL-10 likely indicate residual immune activation despite viral suppression. Neither age nor sex significantly influenced IL-6 or IL-10, indicating that group differences were driven by HIV status and ART duration rather than demographic factors. Similarly, ART regimen class did not influence cytokine levels in this predominantly INSTI-based study population, although a borderline significant effect on IL-6 was observed. A consistent trend toward higher IL-6 levels with increasing viral replication across virological categories was also observed, although statistical significance was not achieved due to an imbalance in group sizes. Collectively, these findings support IL-6 as an indicator of residual immune activation among ART-treated PLHIV with viral suppression in Nigeria. 

Routine HIV care and treatment should continue to prioritise viral load testing as the primary indicator of treatment success and virological control. However, IL-6 monitoring may provide clinically useful supplementary information, particularly among virally suppressed individuals, even when viral loads are undetectable, because standard monitoring may underestimate persistent inflammatory activity. In such patients, IL-6 assessment could help identify residual immune activation that conventional markers may miss.

Healthcare providers should recognise that virological suppression does not necessarily equate to complete immunological normalisation. Persistent elevation of IL-6 in clinically stable ART-treated individuals may reflect ongoing inflammation and should prompt clinicians to assess for underlying non-AIDS-related morbidity, including cardiovascular disease and malignancy. 

Although routine cytokine testing may not yet be feasible in many resource-limited settings due to cost and laboratory constraints, further research on affordable point-of-care cytokine biomarkers should be prioritised to improve long-term HIV care. 

Larger prospective longitudinal studies that incorporate CD4 count data, reservoir size measurements, clinical outcome tracking, and markers of systemic inflammation (CRP, ESR, ferritin, etc.) are needed to confirm these temporal patterns and to define clinically validated cytokine thresholds for use in HIV monitoring protocols across sub-Saharan Africa.

Future clinical trials evaluating the role of adjuvant anti-inflammatory strategies, such as low-dose statins or IL-6 blockade with tocilizumab, in ART-treated PLHIV with persistently elevated IL-6 despite virological suppression would assess whether reducing residual inflammation yields measurable improvements in long-term health outcomes.

## Supplementary Information

Below is the link to the electronic supplementary material.

Supplementary Material 1

## Data Availability

The datasets used and/or analyzed during the current study are available from the corresponding author on reasonable request, subject to ethical considerations and participant confidentiality.

## Abbreviations

ABC: bbreviations
AIDS: Acquired Immunodeficiency Syndrome
ANCOVA: Analysis of Covariance
ANOVA: Analysis of Variance
ART: Antiretroviral Therapy
ATV/r: Atazanavir/Ritonavir
CD4: Cluster of Differentiation 4
CD8: Cluster of Differentiation 8
CRP: C-Reactive Protein
DRV: Darunavir
DTG: Dolutegravir
ELISA: Enzyme-Linked Immunosorbent Assay
ESR: Erythrocyte Sedimentation Rate
HIV: Human Immunodeficiency Virus
HSD: Honestly Significant Difference
IL-6: Interleukin-6
IL-10: Interleukin-10
INSTI: Integrase Strand Transfer Inhibitor
IQR: Interquartile Range
JAK: Janus Kinase
3TC: Lamivudine
NF-kB: Nuclear Factor Kappa-Light-Chain-Enhancer of Activated B Cells
NHREC: National Health Research Ethics Committee
NNRTI: Non-Nucleoside Reverse Transcriptase Inhibitor
NRTI: Nucleoside Reverse Transcriptase Inhibitor
pg/ml: Picograms per Millilitre
PI: Protease Inhibitor
PLHIV: People Living with HIV
PWH: People with HIV
RNA: Ribonucleic Acid
ROC: Receiver Operating Characteristic
SD: Standard Deviation
SEM: Standard Error of the Mean
STAT3: Signal Transducer and Activator of Transcription 3
TDF: Tenofovir Disoproxil Fumarate
TLR: Toll-Like Receptor
TNF-a: Tumour Necrosis Factor Alpha
UPTH: University of Port Harcourt Teaching Hospital
VL: Viral Load
WHO: World Health Organization
