# Supplementary material for: Serum Interleukin-6 and Interleukin-10 levels in people living with HIV on short-term and long-term antiretroviral therapy: a comparative cross-sectional study with HIV-negative controls
Source: BMC Infect Dis. 2026 Jun 27;26:1675. doi: 10.1186/s12879-026-13859-6 (PMC13587376; doi:10.1186/s12879-026-13859-6)
Supplement: Supplementary file 1 [file 12879_2026_13859_MOESM1_ESM.docx]

**UNIVERSITY OF PORT HARCOURT**

*Department of Haematology, Blood Transfusion and Immunology*

**PARTICIPANT CONSENT FORM**

*Study Title: Serum Interleukin-6 and Interleukin-10 Levels in People Living With HIV on Short-Term and Long-Term Antiretroviral Therapy: A Comparative Cross-Sectional Study With HIV-Negative Controls*

**Principal Investigator:** Adegoke Adekunle Ayomide, MSc Student, Department of Immunology, University of Port Harcourt. Tel: 08148660090 | Email: adekunlexyz16@gmail.com

**PURPOSE**

This study aims to measure IL-10 and IL-6 cytokine levels in people living with HIV on ART and compare them with those of healthy individuals. Findings may help doctors better understand immune recovery during HIV treatment and guide future monitoring strategies.

**PROCEDURES**

If you agree to participate: (1) A single 5 mL blood sample will be drawn from your vein. (2) No additional HIV test will be performed. You will be assigned to one of three groups: people living with HIV on short-term ART (<6 months), long-term ART (≥2 years), or HIV-negative healthy controls.

**RISKS**

Risks are minimal and limited to mild discomfort, slight pain, or rare minor bruising/bleeding at the blood collection site. No major risks are anticipated.

**BENEFITS**

You may not directly benefit. However, results may improve understanding of immune recovery in PLHIV on ART and contribute to better treatment and monitoring strategies in the future.

**CONFIDENTIALITY**

All information will be kept strictly confidential. Your name will not appear in any report or publication. Data will be coded and accessible only to the research team. This study has been approved by the Ethics Committee of UPTH (NHREC/UPTHHREC/03/2023).

**VOLUNTARY PARTICIPATION**

Participation is completely voluntary. There is no cost to you. You may refuse or withdraw at any time without any consequences to your medical care.

**CONSENT STATEMENT**

I have read (or had read to me) the information above, had the opportunity to ask questions, and voluntarily agree to participate in this study and to allow the research team access to my relevant medical records.

Participant’s Signature: _____________________________	Date: _______________

Researcher’s Signature: _____________________________	Date: _______________

**UNIVERSITY OF PORT HARCOURT**

*Department of Haematology, Blood Transfusion and Immunology*

**INTERVIEWER-ADMINISTERED QUESTIONNAIRE**

*Study Title: Serum Interleukin-6 and Interleukin-10 Levels in People Living With HIV on Short-Term and Long-Term Antiretroviral Therapy: A Comparative Cross-Sectional Study With HIV-Negative Controls*

**FOR INTERVIEWER USE ONLY**

| Participant ID: ___________ | Date of interview: ___________ | Interviewer's name: ___________ |
| --- | --- | --- |
| Group assigned: A / B / C (circle) | Sample collection time: ___________ |  |

**Section A: Sociodemographic Information**

1. What is your age?

Age: ________________________________________

2. What is your sex?

- Male
- Female

3. What is your marital status?

- Single
- Married
- Divorced
- Widowed

4. What is your current occupation?

Occupation: ________________________________________

**Section B: HIV Diagnosis and ART History**

***Note to interviewer:*** *Questions in this section are for PLHIV (Groups A and B) only. Skip to Section C for HIV-negative controls.*

5. In what year were you diagnosed with HIV?

Year of diagnosis: ________________________________________

6. Are you currently on antiretroviral therapy (ART)?

- Yes
- No

7. If yes, when did you start ART? (Month and year)

Start date (MM/YYYY): ________________________________________

8. What ART medication are you currently taking? (Ask the participant to show or name their drugs if possible)

ART regimen: ________________________________________*.*

9. How regularly do you take your ART medication?

- Every day without missing
- Most days, but sometimes forget
- Irregularly

10. Have you ever stopped taking your ART for more than seven days continuously?

- Yes
- No

If yes, state reason: ___________________________________

**Section C: Clinical and Medical History**

11. Have you been diagnosed with any of the following conditions? (Tick all that apply)

- Tuberculosis (TB)
- Hepatitis B
- Hepatitis C
- Cryptococcal meningitis
- Pneumocystis pneumonia (PCP)
- Any other active infection (specify): ____________________________
- None of the above

12. Are you currently experiencing symptoms of any active infection (such as Hepatitis B or C) or inflammatory illness?

- Yes (specify): ____________________________
- No

13. Have you been diagnosed with any autoimmune condition, such as rheumatoid arthritis, lupus, or similar?

- Yes (specify): ____________________________
- No

14. Are you currently taking any of the following medications? (Tick all that apply)

- Steroids (e.g., prednisolone, dexamethasone)
- Immunosuppressants (e.g., azathioprine, methotrexate)
- Biologics or targeted therapies
- None of the above

15. Have you had any major surgical procedure in the last six months?

- Yes (specify type of surgery and approximate date): ____________________________
- No

16. Are you currently pregnant or do you suspect you may be pregnant?

*(For female participants only)*

- Yes
- No
- Not applicable

17. Do you have any other known medical condition that you are currently being treated for?

- Yes (specify): ____________________________
- No

**Section D: Viral Load Information**

Viral load result: _______________________ copies/ml

Date of viral load test: _______________________

**Section E: CYTOKINE PROFILE**

serum il-6 Result: _______________________

date of test: _______________________

serum il-10 result: _______________________

date of test: _______________________

*Ethics approval: NHREC/UPTHHREC/03/2023 | University of Port Harcourt Teaching Hospital*

*All data collected through this questionnaire will be stored securely and reported only in an aggregate, anonymous form.*
